# Hairy Gels: A Computational Study

**DOI:** 10.3390/gels8120793

**Published:** 2022-12-03

**Authors:** Filip Uhlik, Oleg V. Rud, Oleg V. Borisov, Ekaterina B. Zhulina

**Affiliations:** 1 Department of Physical and Macromolecular Chemistry, Faculty of Science, Charles University, 128 00 Prague, Czech Republic; 2 Institute of Macromolecular Compounds of the Russian Academy of Sciences, 199004 St. Petersburg, Russia; 3 Institut des Sciences Analytiques et de Physico-Chimie Pour l’Environnement et les Matériaux, UMR 5254 CNRS UPPA, CEDEX 9, 64053 Pau, France

**Keywords:** gels, bottlebrush, scaling theory, Monte Carlo, molecular dynamics

## Abstract

We present results of MD and MC simulations of the equilibrium properties of swelling gels with comb-like or bottlebrush subchains and compare them to scaling-theory predictions. In accordance with theory, the simulation results demonstrate that swelling coefficient of the gel increases as a function of the polymerization degree of the main chains and exhibits a very weak maximum (or is virtually constant) as a function of the polymerization degree and grafting density of side chains. The bulk osmotic modulus passes through a shallow minimum as the polymerization degree of the side chains increases. This minimum is attributed to the onset of overlap of side chains belonging to different bottlebrush strands in the swollen gel.

## 1. Introduction

Brush-like macromolecules (molecular brushes) have been extensively studied theoretically and experimentally for a number of decades [1,2,3,4,5,6,7,8,9,10,11]. Both intra- and intermolecular interactions between side chains densely attached to the molecular backbone determine specific conformational and dynamic properties of molecular brushes, as compared to linear analogues. Chemical (covalent) cross-linking of brush-like polymers with subsequent swelling in a good solvent gives rise to the so called “hairy” gels with strands constituted by molecular brushes. The swelling ratios and osmotic moduli of such gels depend in a complex way on the grafting density and polymerization degree of the side chains decorating the network strands [12,13,14,15,16]. Similar structures (“hairy mesogels”) arise upon self-assembly of triblock copolymers with a comb-like or bottlebrush central block and associated terminal blocks [12,17]. In these, physically cross-linked networks the strands are formed by central blocks of copolymer (molecular brushes) that connect neighboring domains of associated blocks (network cross-links).

A molecular brush consists of a linear chain backbone with multiple side chains tethered to it. Three major synthetic approaches: (i) “grafting to” (pre-synthesized side chains are covalently attached to the backbone); (ii) “grafting through” (polymerization of the so-called macromonomers), and (iii) “grafting from” (side chains are polymerized from the backbone as macroinitiator) produced a wide variety of molecular brushes with linear grafts. An additional structural complexity can be introduced through selective gradients in grafting density or block copolymers as side chains [2,18,19].

The possibility to vary architectural parameters of molecular brushes (such as side chain length and grafting density) allows for control and adjustment of static and dynamic properties of both bulk materials and gels thereof [2,6]. Furthermore, branched architectures with bottlebrush motifs, such as barbwire [20,21,22] or dendronized polymers [23,24], have been synthesized and explored theoretically and experimentally [25,26,27]. Combining branched architecture with temperature-, pH-, and light-responsive functions in the main and side chains of molecular brushes opens new opportunities for smart material design [17,28,29,30,31,32,33,34,35]. Since properties of the constituent macromolecules play a governing role in functions of the materials, analytical theories [36,37,38,39,40,41,42] and self-consistent field numerical [43] approaches; and coarse-grained computer simulations [44,45,46,47,48,49,50,51,52,53] of branched polymers have been used to corroborate the relationships between macromolecular architecture and experimentally accessible properties of melts, solutions, and thin films comprising molecular brushes.

It was demonstrated [31] that thermo-responsive triblock copolymers with linear (L) terminal and bottlebrush (B) central blocks can produce hydrogels upon association of L-blocks in spherical domains physically cross-linking B-strands. For example, PNIPAM-bbPEG-PNIPAM triblocks self-assembled upon reaching their lower critical solution temperature (LCST) to produce two types of polymer networks: injectable hydrogels at body temperature and elastomers after water evaporation [31]. The gelation process was attributed to LCST-triggered microphase separation of the PNIPAM L-blocks, and the forming network in both hydrogels and elastomers was homogeneous, in contrast to microphase-separated linear counterparts.

Self-assembly of bottlebrush block copolymers in solutions remains a topic of intensive experimental [54,55,56,57,58,59,60,61,62,63,64] and theoretical [42,65,66] research. It was demonstrated that branched architecture of soluble blocks leads to a variety of self-assembling aggregates that could potentially serve as precursors of gels with bottlebrush strands.

The goal of this paper is to compare the recent theoretical predictions on “hairy” gels with the results of MD and MC computer simulations. In Section 2, we briefly review the scaling model of hairy gels, and summarize the theoretical predictions on its swelling behavior and mechanical properties. We then compare the theoretical predictions with the data from Monte Carlo (MC) and molecular dynamic (MD) simulations. In Section 4, we present the details of implemented MC and MD methods. In Section 3, we formulate conclusions and outline perspectives for further development in theory and modeling of bottlebrush architectures.

## 2. Results and Discussion

### 2.1. “Hairy” Polymer Gel: Scaling Model

**Figure 1:**
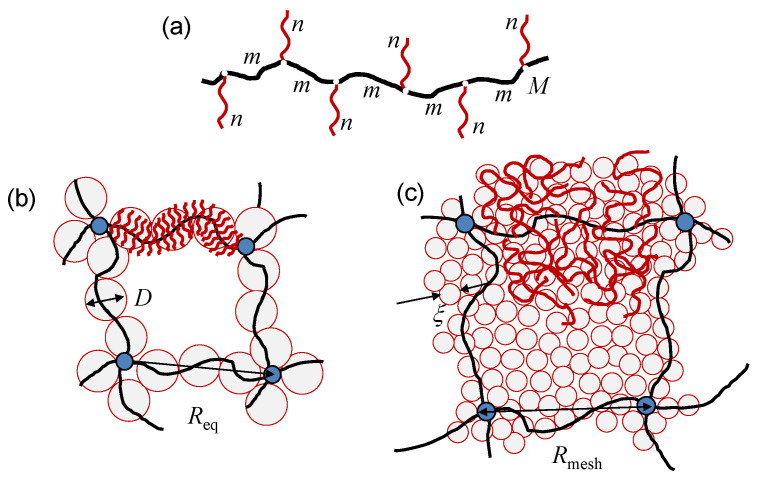
Schematics of graft-polymer, with {M,n,m} being the DPs of the main and side chains and of the spacers, respectively. (**a**) Hairy gel with bottlebrush strands in hollow mesh (**b**) and filled mesh (**c**) regimes. Backbones of strands are colored in black, side chains are in red, and cross-links are marked as blue circles. $R_{eq}$ is the equilibrium end-to-end distance of the main chains of the gel strands (the mesh size). Superblobs with size *D* (hollow-mesh regime) and concentration blobs with size ξ<D in semi-dilute solution of side chains (filled-mesh regime) are shaded light gray.

Swelling of chemically and/or physically cross-linked networks with brush-like strands in a good solvent yields “hairy” gels. Each strand in a hairy gel constitutes a molecular brush with degree of polymerization (DP) *M* in its flexible backbone (main chain), and equally flexible spacers and side chains with DPs *m* and *n*, respectively (see Figure 1a). In contrast to networks with linear strands, the swelling and elastic properties of hairy gels depend not only on the cross-linking density defined by the strand DP N=M(1+n/m), but also on *n* and grafting density (1/m) of side chains at a given *M*. If n>m, side chains overlap and stretch normally to the backbone due to monomer–monomer interactions, giving rise to a bottlebrush (molecular brush). If n<m, the side chains exhibit coil conformations, making the polymer comb-like.

A molecular brush is envisioned as a wormlike chain with thickness *D* (which is end-to-end distance of the side chains normally to the backbone), spacer end-to-end distance *h*, effective contour length L≅Mh/m, and persistence length $l_{p}≃D$. In the framework of scaling model [36,42], the strand thickness *D* and spacer end-to-end distance *h* are specified by the balance between the elastic stretching of side chains and spacers, and repulsive monomer-monomer interactions in the cylindrical layer around the main chain, to give

(1) $$D/a≅\left\{\begin{array}{cc} n^{\nu }{\left(n/m\right)}^{\nu (1-\nu )/2}, & m\geq m^{*}\\ n^{2\nu /(1+\nu )}m^{-(1-\nu )/(1+\nu )}, & m\leq m^{*} \end{array}\right.$$

(2) $$h/a≅\left\{\begin{array}{cc} m^{\nu }{\left(n/m\right)}^{\nu (1-\nu )/2}, & m\geq m^{*}\\ m, & m\leq m^{*} \end{array}\right.$$

The first and the second lines in Equations (1) and (2) correspond to partial and close to maximal stretching of spacers in the main chain, respectively (with $m^{*}≅n^{\nu /(2+\nu )}$ separating two elasticity regimes for the backbone); *a* is the length of monomer unit in the backbone and side chain; and ν is Flory exponent (ν≈3/5 in good solvent, and ν=1/2 in theta-solvent). Notably, the scaling relations in Equations (1) and (2) have asymptotic character—that is, they apply only for n≫1, n≫m, and $M\gg M^{*}$ with

$$M^{*}≃\left\{\begin{array}{cc} m^{1-\nu }n^{\nu }, & m\geq m^{*}\\ n^{2\nu /(1+\nu )}m^{-(1-\nu )/(1+\nu )}, & m\leq m^{*} \end{array}\right.$$

In scaling terms, $M≃M^{*}$ (or, equivalently L≃D) separates bottlebrushes with L≫D from starlike polymers with L≪D. In realistic experimental and simulation systems, $n\leq 10^{2}$ and m≃1. Due to the local cylindrical symmetry, a strong overlap of moderately long side chains occurs only close to the backbone, and therefore the scaling dependences predicted by Equations (1) and (2) are only approached in the currently attainable range of *n*, m, and *M*. However, due to still noticeable stretching of the side chains normally to the backbone ($D>an^{\nu }$), a bottlebrush molecule behaves as a self-avoiding chain composed of L/D impermeable subunits (“superblobs” with size *D* each), and its overall size $R_{eq\;}$ in dilute solution scales as

(3) $$R_{eq}≅D{\left(\frac{L}{D}\right)}^{3/5}={\left(\frac{Mh}{ma}\right)}^{3/5}{\left(\frac{D}{a}\right)}^{2/5}$$

Distribution of polymer’s density within a swollen hairy gel could be inhomogenuous. Two structural regimes, hollow mesh and filled mesh gel, are distinguished depending on the ratio between the mesh size, $R_{mesh\;}$, and the superblob size, *D* [15,16]. In the hollow mesh regime (Figure 1b), each strand constitutes a molecular brush with thickness $D\ll R_{mesh}$, and weak overlap of neighboring strands ensures hollow space (mesh) between cross-links. In this case, the mesh size $R_{mesh}$ can be evaluated using the $c^{*}$-theorem of de Gennes, [67] to give $R_{mesh}≃R_{eq}$. In the filled-mesh regime (Figure 1c), the neighboring strands strongly overlap, giving rise to a semi-dilute solution of side chains with an almost uniform concentration $c≃Na^{3}/R_{mesh}^{3}$. The interior of the gel is envisioned as a closely-packed array of the concentration blobs with size $\xi \left(c\right)∼ac^{-\nu /(3\nu -1)}$<D. In this case, the equilibrium mesh size $R_{mesh}$ results from the balance between gel osmotic pressure, $\pi /k_{B}T≅\xi^{-3}\left(c\right)$, and the conformational elasticity of the backbones (renormalized according to the polymer concentration *c* inside the gel).

**Figure 2:**
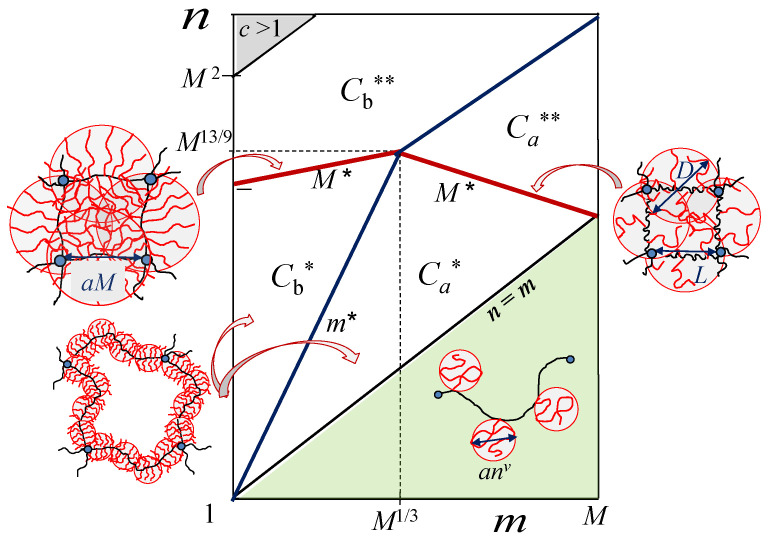
Scaling-type diagram of states for hairy gel in {n,m} coordinates in good solvent (ν=3/5). In the hollow-mesh regimes $C_{b}^{*}$ and $C_{a}^{*}$, bottlebrush strands have either partially or almost fully stretched spacers, respectively. In filled-mesh regimes $C_{b}^{**}$ and $C_{a}^{**}$, the interior of the gel constitutes a semi-dilute solution of side chains. Schematics demonstrate strand conformations in regimes $C_{b}^{*}$ and $C_{a}^{*}$, and at boundaries $C_{b}^{*}-C_{b}^{**}$ and $C_{a}^{*}-C_{a}^{**}$ (that correspond to backbone length $M=M^{*}$ at which bottlebrush transforms in starlike polymer with L=D). Boundary $C_{b}^{*}-C_{a}^{*}$ (marked $m^{*}$) separates bottlebrush strands with fully ($m<m^{*}$) and partically ($m>m^{*}$) stretched spacers. In the shaded green area, strands are comb-like (side chains are unstretched coils). Strands in regimes of semi-dilute solutions $C_{b}^{**}$ and $C_{a}^{**}$ are shown in Figure 1.

To facilitate comparison between the theoretical predictions and the simulation data, we present in Figure 2 the scaling-type diagram of states for hairy gels [16] in n,m log–log coordinates (with ν=3/5). In the hollow-mesh regimes $C_{a}^{*}$ and $C_{b}^{*}$, each spacer in the main chain is, respectively, partially or almost fully stretched. The boundary $C_{a}^{*}-C_{b}^{*}$ corresponds thereby to m=$m^{*}$, indicating the onset of spacer strong stretching in individual strands at given n. Similar situation occurs in the filled mesh regimes $C_{a}^{**}$ and $C_{b}^{**}$. That is, the boundary $C_{a}^{**}-C_{b}^{**}$ corresponds to the onset of spacer strong stretching in the filled-mesh regimes (semi-dilute solutions of the side chains). At the boundaries $C_{b}^{*}-C_{b}^{**}$ and $C_{a}^{*}-C_{a}^{**\;}$ (red lines in Figure 2), DP *M* of the backbone becomes equal (in scaling terms) to $M^{*}$, separating bottlebrush and starlike conformations of strands. At these boundaries (indicated by $M^{*}$), each strand comprises on the order of one superblob with size *D* (L≃D), as schematically shown in Figure 2.

The scaling expressions for the equilibrium mesh size $R_{mesh}/aM^{\nu }$, gel swelling coefficient (that is, ratio of volumes *V* in the swollen and dry states)

(4) $$Q=\frac{V}{V_{dry}}≃\frac{R_{mesh}^{3}}{a^{3}N}=\frac{R_{mesh}^{3}}{a^{3}M}\cdot \left(\frac{m}{n}\right),$$

with N=M(1+n/m) and n≫m, and osmotic bulk modulus

(5) $$\frac{G}{k_{B}T}=c{\left(\frac{\partial \pi }{\partial c}\right)}_{c=c_{eq}}≃\left\{\begin{array}{cc} R_{mesh}^{-3} & \mathrm{hollow}\;\mathrm{mesh}\;\mathrm{regimes}\;C_{a}^{*},\;C_{b}^{*}\\ \xi {\left(c\right)}^{-3} & \mathrm{filled}\;\mathrm{mesh}\;\mathrm{regimes}\;C_{a}^{**},\;C_{b}^{**} \end{array}\right.$$

are collected in Table 1 with corresponding exponents specified for ν=3/5.

As follows from Table 1, an increase in DP *n* of the side chains at fixed DP *M* of the backbone leads to the monotonous increase in mesh size, $R_{mesh}∼n^{\beta }$, in regimes $C_{a}^{*}$ and $C_{a}^{**}$ (exponent β=9/25=0.36), $C_{b}^{*}$ (exponent β=3/10), approaching full extension, $R_{mesh}∼aM$, in regime $C_{b}^{**\;}$ (exponent β=0). At the same time, both swelling ratio *Q* and osmotic bulk modulus *G* exhibit non-monotonic dependences on *n*. Swelling coefficient $Q\left(n\right)∼n^{3\beta -1}$ passes through a maximum at the boundaries $C_{a}^{*}-C_{b}^{*}$ and $C_{a}^{**}-C_{b}^{**}$, and osmotic modulus G(n) passes through a minimum upon crossing the boundaries of regime $C_{b}^{**}$. The maximum in Q(n) dependence is weak due to a small value of the exponent which changes from +0.08 to −0.1 at the $C_{a}^{*}-C_{b}^{*}$ boundary, and this maximum could even disappear if the apparent exponent $\beta_{app}<1/3$. Recall that the values of exponents in Table 1 were calculated in the limit of n/m≫1. The predicted sharp (jumpwise) minimum in G(n) at the boundaries of regime $C_{b}^{**}$ could be smoothed in computer simulations, leading to shift in the minimum location far left (to smaller values of *n*).

### 2.2. Computer Simulations of Hairy Gel

**Figure 3:**
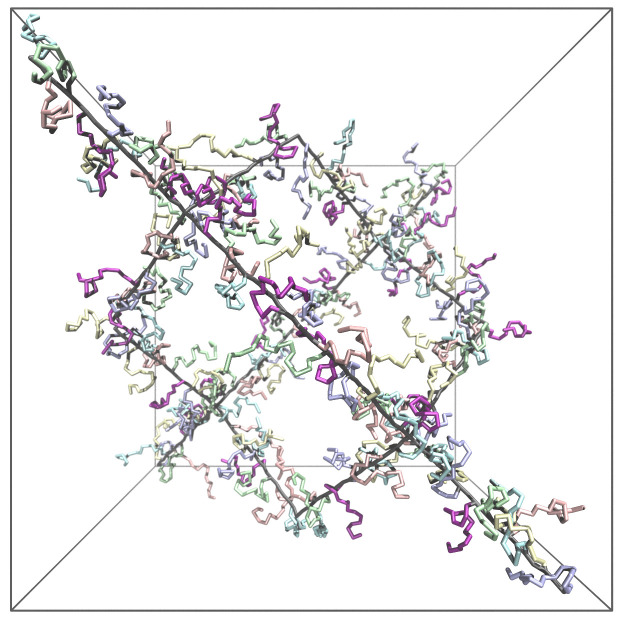
A view of a simulation unit cell with gel backbone (in black color, length M=19) and side chains (in various light colors for clarity, length n=16) grafted every second monomer unit (m=2) intentionally in a state with pressure *P* < 0 (volume *V* greater than in free swelling equilibrium with pressure P=0) to avoid excessive overlapping of side-chains and clearly showing the gel branching. Backbones are indicated in black with side chains in various colors (to avoid crowding).

We used both MC and MD simulations to explore the equilibrium-swelling behavior of hairy gels in a good solvent (ν=3/5). In Figure 3, we present the simulation box which has the same geometry in MC and MD simulations. That is, 16 polymer chains were connected to a diamond-like network by eight tetrafunctional cross-linking units, and the network element was put into cubic simulation box of the volume *V* with periodic boundary conditions to emulate an infinite polymer network. It is worth mentioning that MD and MC simulations were performed in NVT and NPT (with pressure P=0 and *V* fluctuating) ensembles, respectively.

**Figure 4:**
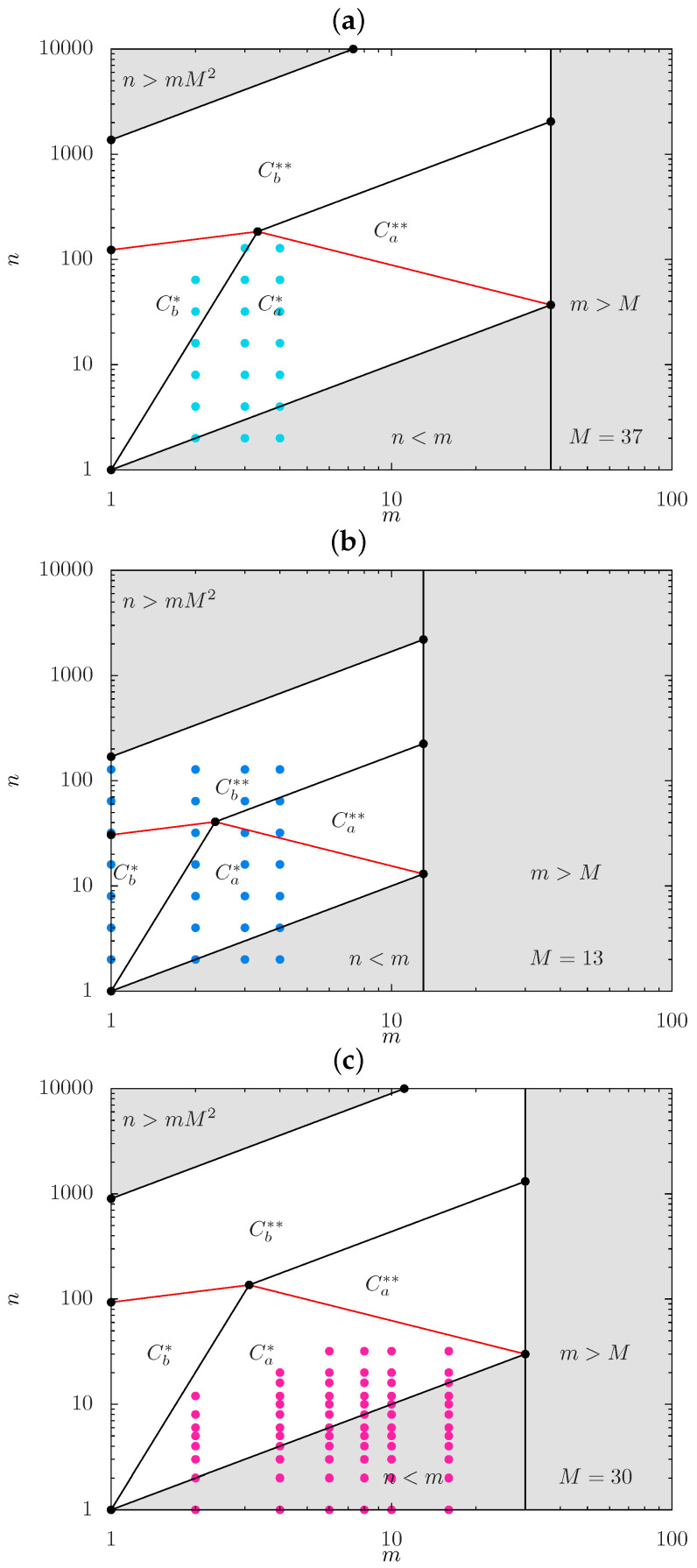
Scaling-type diagrams with positions of boundaries calculated specifically for DP M=37 (**a**) and DP M=13 (**b**) of the backbone, respectively, together with ordered set of symbols {n,m} marking the architectural parameters of bottlebrush strands modeled in MC simulations and (**c**) for M=30 in MD simulations.Shaded area with $n\geq mM^{2}$ corresponds to unphysical values of c≥1.

In Figure 4a,b, we present the scaling-type diagrams with positions of the boundaries calculated specifically for DP M=37 and DP M=13 of the backbone, respectively, together with set of symbols {n,m} marking the architectural parameters of bottlebrush strands modeled in MC simulations. In Figure 4c, a similar diagram is presented for M=30 with set of symbols {n,m} marking the parameters of strands modeled in MD simulations.

While positions of the boundaries in Figure 4 are specified with accuracy of the numerical prefactors on the order of unity, it is still expected that MC simulations for M=37 (Figure 4a) with small values of 1≤m≤4 and increasing *n* (up to n=128) cover regimes $C_{a}^{*}$ and $C_{b}^{*}$. In contrast, Figure 4b indicates that for M=13, MC data pass through regimes $C_{a}^{*}$$C_{b}^{*}$, and even enters regime $C_{b}^{**}$. According to Figure 4c, MD simulations cover a considerably larger interval of *m*-values (up to m=50). However, many of the symbols correspond to comb-like strands (area with n/m<1, shaded gray), and the rest of the data probes only regime $C_{a}^{*}$.

#### 2.2.1. Average End-to-End Distances of Strands and Side Chains

**Figure 5:**
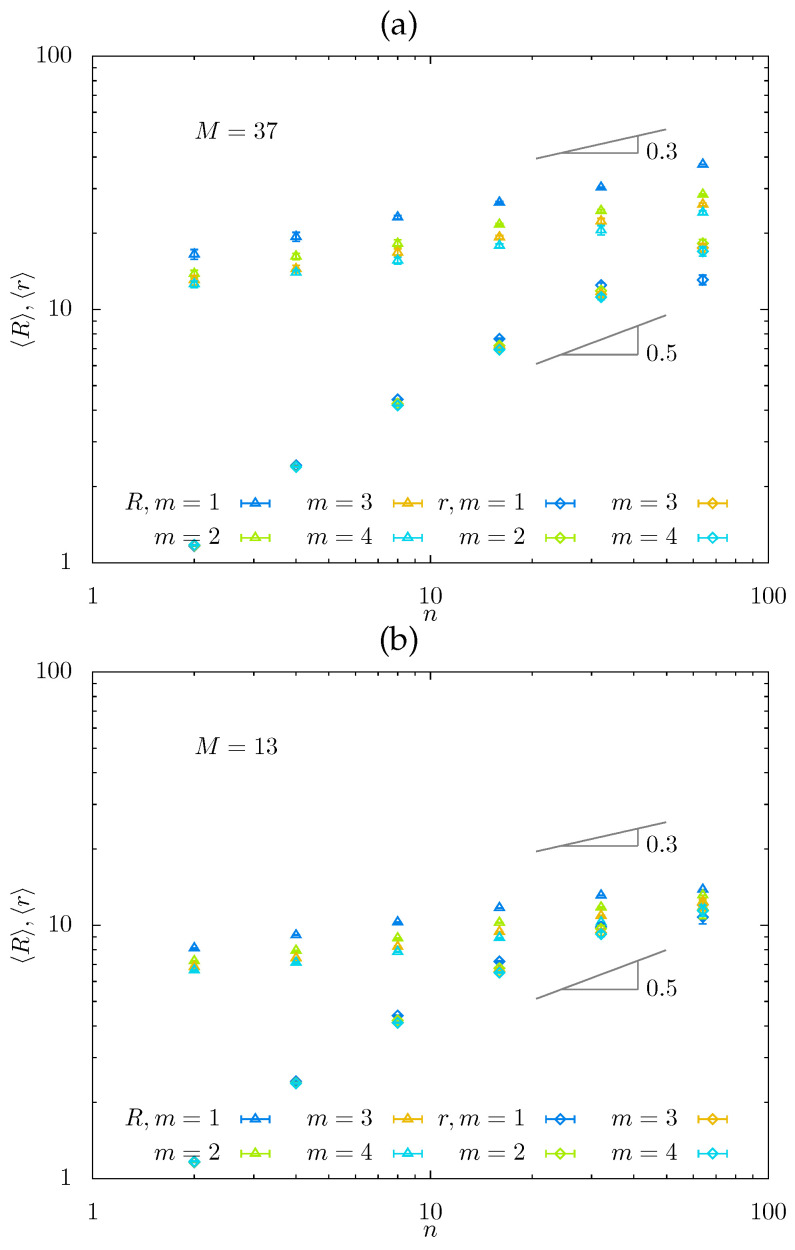

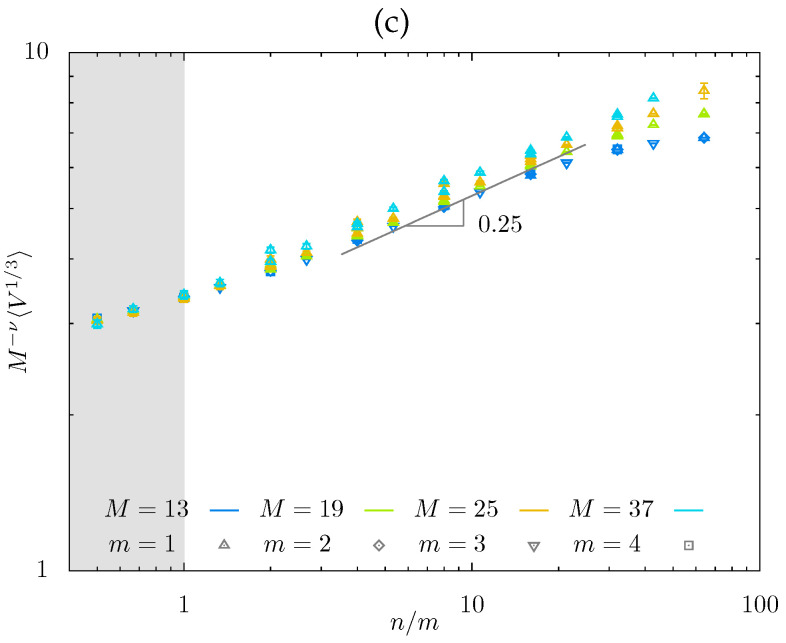
Average mesh size $R={\langle V\rangle }^{1/3}$ and end-to-end distances of the side chains 〈r〉 as a function of *n* for m=1,2,3,4 for M=13 (**a**) and M=37 (**b**) obtained from MC simulations. In panel (**c**), the mesh size is normalized by $M^{\nu }$. Here and below, the gray area corresponds to comb-like strands with n/m<1.

In Figure 5, we plot the average mesh size $R_{mesh}≃{\langle V\rangle }^{1/3}$ of a free-swelling gel, and the average end-to-end distance $\langle r\rangle ≃r_{sc\;}≃D$ of the side chains obtained in MC simulations as a function of *n* for m=1,2,3, and 4 in log–log coordinates.

At n≳10 (when the side chains stretch normally to the backbone), different *m*-values produce almost parallel lines in $R_{mesh}\left(n\right)∼n^{\beta }$ dependences, with apparent slopes $\beta_{app}=0.22-0.25$, i.e., smaller than the theoretically predicted β (i.e., 0.36 in regime $C_{a}^{*}$ and 0.30 in regime $C_{b}^{*}$). For $r_{sc}\left(n\right)∼n^{\alpha }$, the correspondence between the theoretical exponents α (i.e., 0.75 in regimes $C_{b}^{*}$ and 0.72 in regime $C_{a}^{*}$) and apparent slopes is expectably [50] worse, consistent with smaller apparent values of $\beta_{app}$. Notably, at M=37, $R_{mesh}\left(n\right)$, and $r_{sc}\left(n\right)$ do not intersect at fixed *m* at any considered *n* (see Figure 5a), indicating that the hairy gel remains in the hollow mesh state. In contrast, at M=13, $R_{mesh}\left(n\right)$ and $r_{sc}\left(n\right)$ become close at the largest values of *n* (see Figure 5b), indicating that the hairy gel approaches the filled-mesh state. In Figure 5c, we plot normalized mesh size $R_{mesh}/aM^{3/5}$ as a function of (n/m) for M=13,19,25, and 37 to demonstrate how MC data collapse on mastercurve with slope $\beta_{app}=0.25$, with maximal deviations for the smallest value of M=13 (presumably approaching regime $C_{b}^{**}$ with $R_{mesh}/aM^{3/5}∼{\left(n/m\right)}^{0}$).

**Figure 6:**
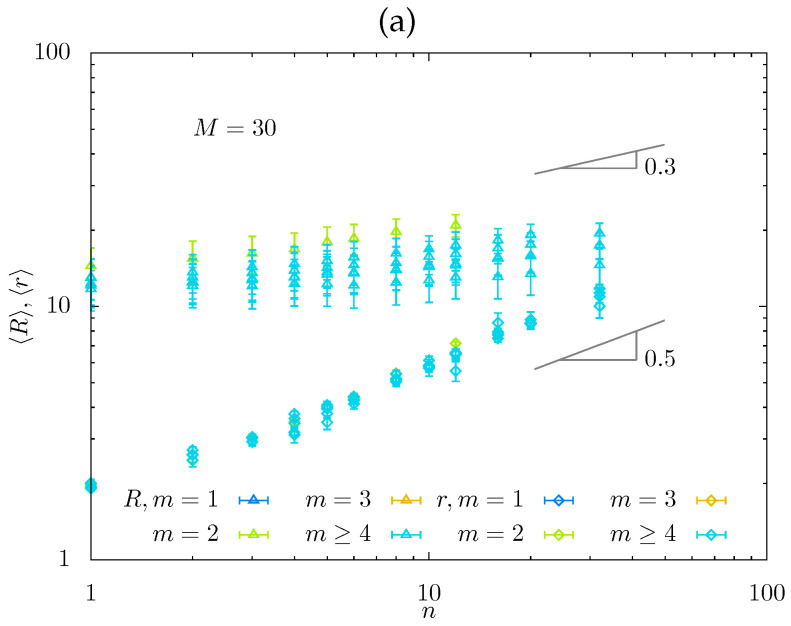

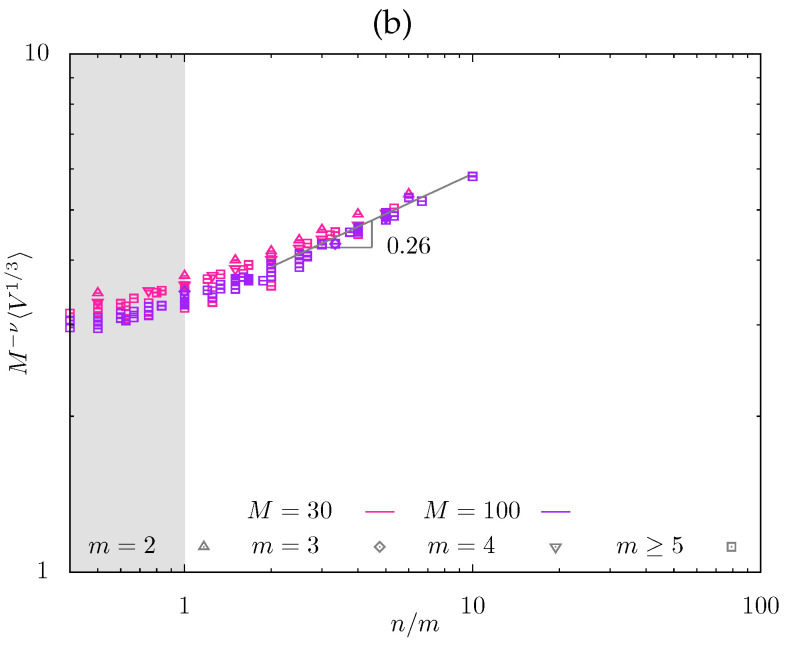
Equilibrium mesh size from MD simulations, $R_{mesh}\left(n\right)∼V^{1/3}$, as a function of *n* for a series of *m*-values and fixed M=30 (**a**), and normalized mesh size $R_{mesh}\left(n\right)M^{-3/5}$ versus n/m with slope $\beta_{app}=0.26$ (**b**).

In Figure 6, the equilibrium strand end-to-end distance $R_{mesh}\left(n\right)∼<V{>}^{1/3}$ is presented for a series of *m*-values and fixed M=30, as obtained from MD simulations. While values of n<m correspond to comb-like strands, the data for relatively small *m* and n>10 are used in Figure 6 to evaluate apparent exponent $\beta_{app}$ in the dependence $R_{mesh}\left(n\right)M^{-3/5}∼{\left(n/m\right)}^{\beta_{app}}$ to give $\beta_{app}\approx 0.26$, in accordance with the results of MC simulations.

#### 2.2.2. Gel Swelling Coefficient

**Figure 7:**
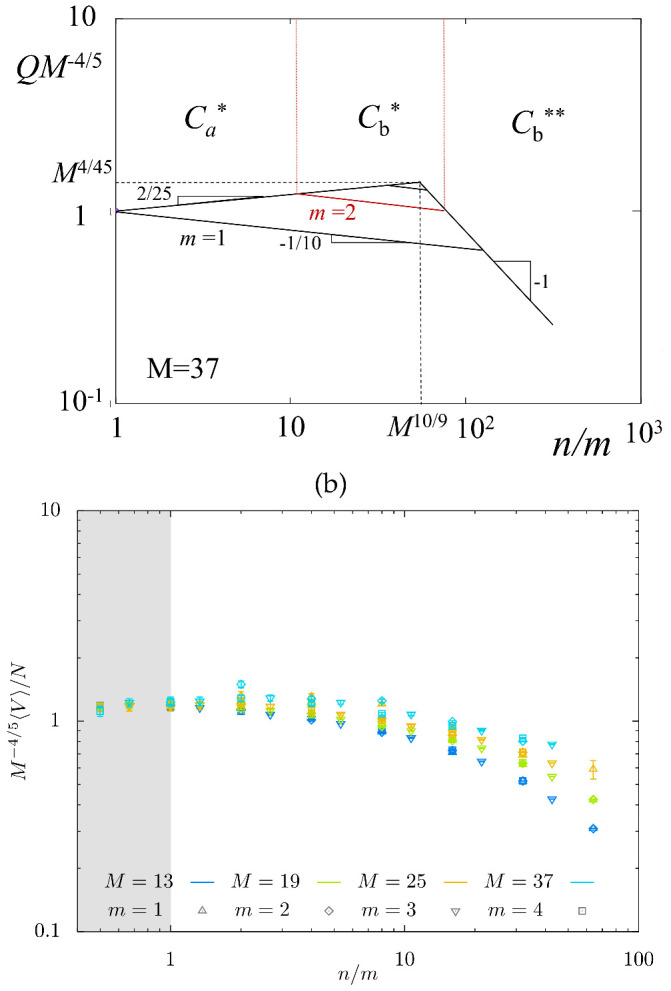
Theoretical (**a**) and MC-simulated (**b**) swelling coefficient *Q* as a function of n/m. Scaling dependences for theoretical *Q* (with all numerical coefficients equal to unity) were calculated for M=37. $C_{a}^{*}-C_{b}^{*}$ and $C_{b}^{*}-C_{b}^{**}$ boundaries are indicated for m=2.

In Figure 7, we present the normalized equilibrium swelling coefficient $QM^{-4/5}=VM^{-4/5}/a^{3}N$ as a function of n/m predicted by the scaling model (Figure 7a) and obtained from MC simulations (Figure 7b) in log–log coordinates. The power-law dependences $QM^{-4/5}$ in Figure 7a were calculated for M=37 with slopes indicated in Table 1 (that is, 2/25,−1/10, and −1 in regimes $C_{a}^{*},C_{b}^{*},C_{b}^{**}$, respectively) and m=1,2,3, with all numerical prefactors assigned unity. For m=2, *Q* is shown by red lines; for m≥4 only regimes $C_{a}^{*},C_{b}^{**}$ are feasible. The maximum predicted for M=37 corresponds to $QM^{-4/5}$≈1.38. A decrease in *M* shifts location of the maximum to the left and makes it less pronounced.

MC simulations data in Figure 7b show the dependence of $QM^{-4/5}$ on n/m for series of *M*-values. The predicted weak maximum is not well pronounced; mostly, its decreasing (right) branch is seen in MC simulations. As strands with M=37 are not expected to enter regime $C_{b}^{**}$ (see Figure 4a), the slope dQ/dn is far from the predicted exponent −1. However, for smaller M=13 for which regime $C_{b}^{**}$ is feasible (see Figure 4b), the slope of MC data approaches −1, as predicted.

**Figure 8:**
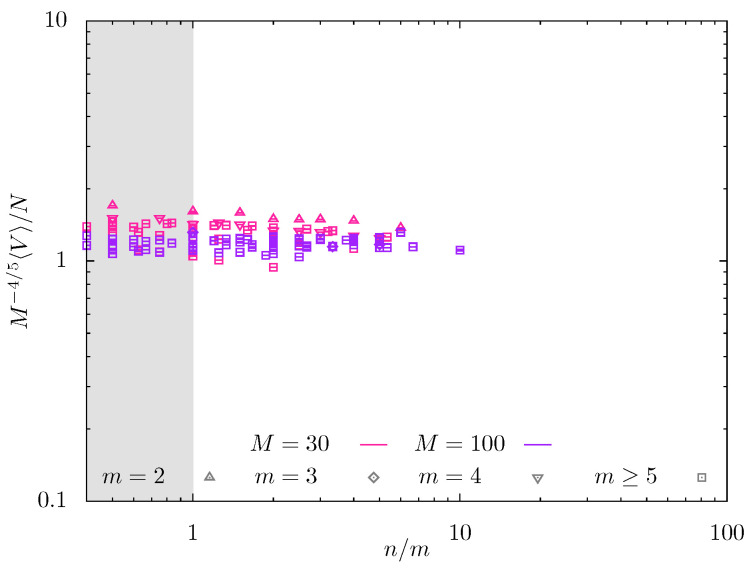
Normalized swelling coefficient $QM^{-4/5}$ from MD simulations for M=30 (red symbols) and 100 (violet symbols) as a function of n/m.

The data from MD simulations in Figure 8 also indicate weak dependence of swelling coefficient *Q* on n/m. Here, the normalized swelling coefficient $QM^{-4/5}$ is presented as a function of n/m for a wider variety of *m*-values and two values of M=30 (red symbols) and M=100 (violet symbols), and probing regime $C_{a}^{*}$ in which the predicted slope is 2/25=0.08. For M=30, the data in Figure 8 with different *m*-values remain rather scattered; the increase in backbone DP *M* up to M=100 decreases the scattering of the data that collapse on mastercurve with close to zero slope. Notably, the numerical prefactor in $QM^{-4/5}$ versus n/m dependences is close to unity in both MC and MD simulations, consistent with the scaling model.

#### 2.2.3. Osmotic Bulk Modulus

**Figure 9:**
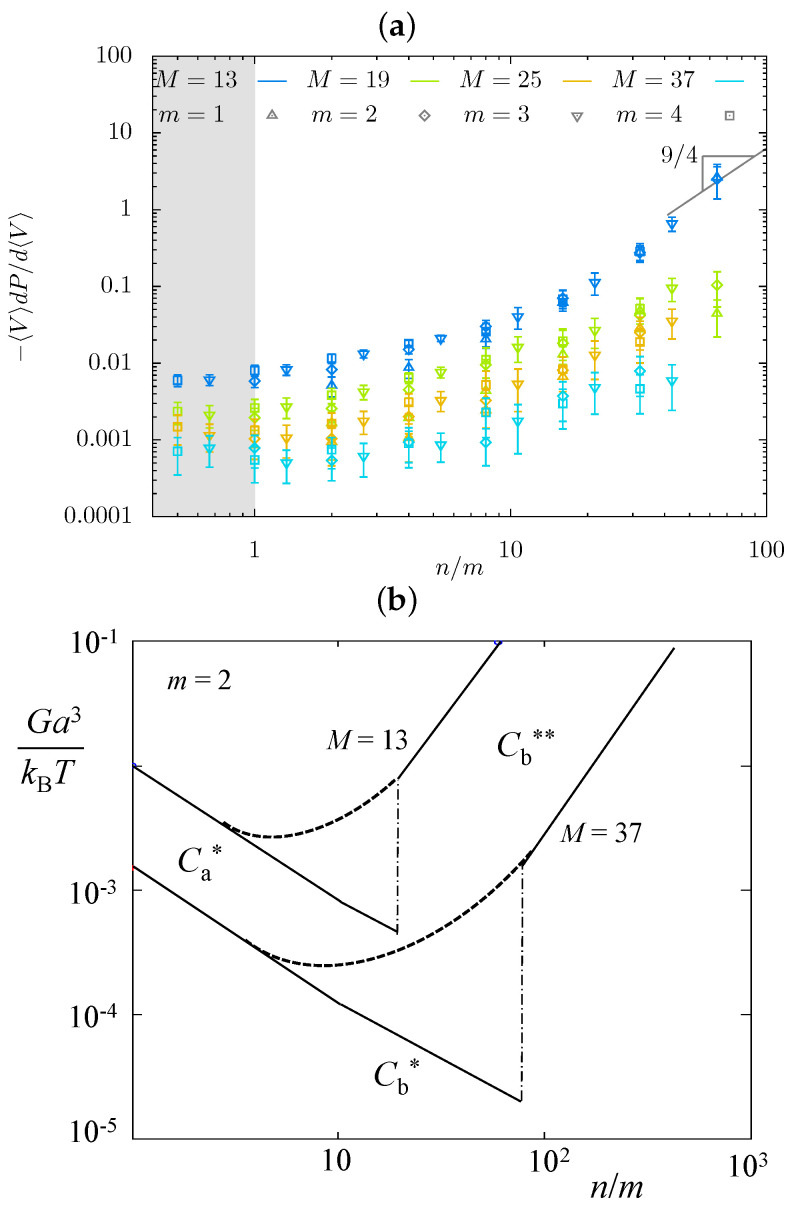
Osmotic modulus *G* as a function of n/m in MC simulations (**a**) and scaling model (**b**). A conjecture that sharp minimum could be smoothed in simulations is illustrated by the dashed lines (**b**).

In Figure 9a we present the MC data for the gel osmotic bulk modulus, $G=c{\left(\partial \pi /\partial c\right)}_{c=c_{eq}}$. Here, π(c) is osmotic pressure, and $c_{eq}≃Q^{-1}\;$ is the equilibrium concentration of monomer units in the hairy gel. As is seen in Figure 9a, the osmotic modulus *G* decreases with increasing DP *M* of the strand backbone, in qualitative agreement with the theoretical predictions in Table 1. However, the predicted minimum in G(n) dependence is not detected in MC simulations. An increase in *M* flattens G(n) dependence, only pointing at the possibility of minimum formation.

A conjecture is that the sharp minimum predicted by the scaling model could be smoothed in MC simulations, as schematically illustrated by the dashed lines in Figure 9b. Another factor is the effect of perturbed dense cylindrical layers circumventing the backbones. Although Figure 9b is merely schematic and does not specify the shape or position of the dotted lines, it is clear that the theoretically predicted exponents for G(n) would not work in the smoothed region, and therefore, collapse of *G*-data on the mastercurve is not expected. However, this schematic indicates that minimum in G(n) dependence can move left and fall out of the considered ranges of the strand architectural parameters.

## 3. Conclusions

Based on the scaling description of molecular brushes, the theory specifies power-law dependences for the equilibrium properties of “hairy” gels with bottlebrush strands. Four structural regimes of hairy gels were distinguished: two hollow-mesh regimes, $C_{a}^{*}$ and $C_{b}^{*}$, with partially and almost fully stretched spacers; and two filled mesh regimes, $C_{a}^{**}$ and $C_{b}^{**}$, with partially or almost fully stretched backbones embedded in a semi-dilute solution of side chains. Two supplementary computer simulation techniques, MC and MD, were used to probe the structure of free-swelling in good solvent gel with varied strand architectural parameters {M,n,m} to corroborate the theoretical model. It was demonstrated that MC simulations of hairy gels with short spacers (m≤4) could cover regimes $C_{a}^{*}$ and $C_{b}^{*}$, and approach regime $C_{b}^{**}$. MD simulations of gels with a wider variety of *m*-values could probe regime $C_{a}^{*}$. In all cases, limited extension of the gel regimes (Figure 4) and restriction n/m≫1 make it challenging to compare the predicted asymptotic values of exponents (Table 1) with apparent exponents from MC and MD simulations. In addition, it is not surprising that apparent exponents deviate from the theoretical ones for the considered set of strand parameters {M,n,m}.

According to the scaling model, the gel mesh size $R_{mesh}∼M^{\nu }n^{\beta }$ increases monotonously with increasing of both the DP *n* of the side chain and the DP *M* of the strand backbone. The apparent exponent $\beta_{app}\approx 0.25-0.26$ estimated from MC and MD simulations was smaller but reasonably close to the theoretical values, β=0.30−0.36. Exponent ν was close to 3/5 as expected for good solvent conditions. The thickness *D* of the bottlebrush strand increased with *n*. However, an approach to the theoretical exponent 3/4 requires $n≳10^{3\;}$ (i.e., much larger *n*-values than have been implemented in MC simulations), and the correspondence between apparent and scaling exponents in D(n) dependence was poor.

The theory predicts that the swelling ratio *Q* of the hairy gels is controlled primarily by the DP *M* of the strand backbone, whereas the dependence on the DP *n* and grafting density 1/m of the side chains is weak. This prediction is in agreement with the MC and MD simulation data. The theory also predicts non-monotonic dependences of osmotic modulus *G* and swelling ratio *Q* of hairy gels on DP *n* of the side chains. The osmotic modulus *G* passes through a minimum corresponding to the overlap threshold of the side chains emanating from different strands. In contrast, swelling ratio *Q* passes through a maximum upon onset of strong stretching of spacers in nonoverlaping strands. The MC computer simulations did not find pronounced extrema in Q(n/m) and G(n/m) dependences and demonstrated only the decreasing branch in *Q*-dependence (with slope approaching −1, predicted in regime $C_{b}^{**}$) and the increasing branch in *G*-dependence (with slope approaching 9/4, predicted in regime $C_{b}^{**}$). Lack of maximum in Q(n) dependence can be explained by the values of apparent exponent $\beta_{app}<1/3$ in $R_{mesh}∼n^{\beta_{app}}$ dependence that automatically eliminates the increasing branch in $Q=R_{mesh}^{3}/a^{3}N$$∼n^{3\beta_{app}-1}$. The relatively small values of $\beta_{app}$ could follow from the limited extension of the scaling regimes for currently considered set of {M,n,m} parameters. A wider set of architectural parameters {M,n,m} is desirable to confront the scaling model of hairy gel with better accuracy.

## 4. Materials and Methods

The model of the gel as a network of 16 linear polymer chains, each consisting of *M* monomer units. These polymer chains are connected to a diamond-like network by eight cross-linking units. The side chains of the lenght *n* are grafted on each *m*-th monomer of the main chain (see Figure 3). The network was put into cubic simulation box of the volume $V_{gel}$ with periodic boundary conditions, which virtually emulates an infinite polymer network.

### 4.1. Monte Carlo Simulations

In Monte Carlo simulations, we used a variant of the Hamiltonian (originally called hybrid [68]) Monte Carlo (HMC) method and coarse-grained (CG) models. The HMC uses Hamiltonian dynamics to sample probability distribution $\exp(-H/k_{B}T)$, where H(p,q) is the Hamiltonian, *q* are generalized coordinates, and *p* are generalized momenta. In the simplest case, a separable Hamiltonian H(p,q)=K(q)+V(p) is used, where *K* is kinetic energy and *V* is potential energy for sampling Boltzmann distribution $\exp(-V\left(q\right)/k_{B}T)$. The momenta *p* can be sampled directly, and Hamiltonian dynamics $\dot{q}=\partial H/\partial p$, $\dot{p}=-\partial H/\partial q$ are followed for some time to prepare a new proposal for a Metropolis step [69] with acceptance probability $P_{\mathrm{acc}}=\min\left(1,\exp\left(-\Delta H/k_{B}T\right)\right)$. For the exact dynamics ΔH, it would be zero (time independent Hamiltonian is conserved), and all proposals would be accepted. In practice, instead of exact dynamics, we must use an approximate numerical evolution with numerical integrators. Fortunately, time-reversible, phase-space volume-preserving integrators are available, and their procedure is exact, and any bias due to approximate dynamics is then removed by Metropolis rejections. Moreover, the Hamiltonian for dynamic evolution can be different from the targeted one. This allows for great variability of the method. In the case of polymer simulations, a big improvement can be achieved by a suitable transformation of variables. The potential energy for the standard Rouse (harmonic) model is diagonal in bond vector coordinates $r_{i}-r_{i-i}$. With the freedom of choosing the evolution Hamiltonian, we use new canonically conjugated momenta with bond vector coordinates, and it allows for sampling all normal modes at the same rate [70]. For other potentials and approximate integrators, the method somewhat deteriorates, but it is still much better than simple molecular dynamics and leads to a smaller scaling exponent of the integrated autocorrelation time of the end-to-end distance with chain length, and thus is arbitrarily faster.

In our CG model we use two types of interactions, bonding and non-bonding. For the bonding one, we use a variant of finitely extensible nonlinear elastic (FENE) potential [71] in a form $u_{F}\left(r\right)=-ek_{B}T\log\left(\left(r_{0}+d-x\right)\left(x-r_{0}+d\right)/d^{2}\right)$ for $r\in (r_{0}-d,r_{0}+d)$ and infinite elsewhere, with mild singularities at $r_{0}\pm d$. It is a relatively small complication for simple molecular dynamics where small time-steps are used that potential does not exist outside $\left(r_{0}-d,r_{0}+d\right)$. In HMC, we use the advantage of much longer time-steps that could lead to stepping out of the definition interval. We resolve this in the spirit of HMC and prepare a new potential finite, everywhere being $u_{F}\left(r\right)$ on $\left(r_{0}-d+\epsilon ,r_{0}+d-\epsilon \right)$ and $v\left(r\right)+u\left(r_{0}+d-\epsilon \right)-v\left(r_{0}+d-\epsilon \right)$, where $v\left(r\right)=ek_{B}T{\left(r_{0}/d\right)}^{2}{\left(r/r_{0}-1\right)}^{2}$ elsewhere that is used for dynamic evolution in HMC and the original $u_{F}\left(r\right)$ is used for the Metropolis step. By this combination, we achieve the exact sampling with $u_{F}\left(r\right)$ while avoiding numerical problems. For non-bonding potential, we use soft repulsive potential $u_{S}=e{\left(\left(r-c\right)/r\right)}^{2}$ for r∈(0,c) and 0 elsewhere, where e>0 is an energy parameter and *c* is a cutoff. The potential is smooth at cutoff. Frequently, the standard Lennard–Jones potential [72], originally suggested for simulations of neon, is used for this purpose. Although the $1/r^{6}$ part can be justified for two atoms (with at least one in an *S*-state) at very long distances, the $1/r^{12}$ part lacks such a simple justification, the less for the potential that should represent CG macromolecular system in a solution. By taking a systematically coarse-grained model based on all-atom empirical force-field and fitting the corresponding part for small distances, we find a much smaller exponent [73] of about 2, as for our u(r).

**Figure 10:**
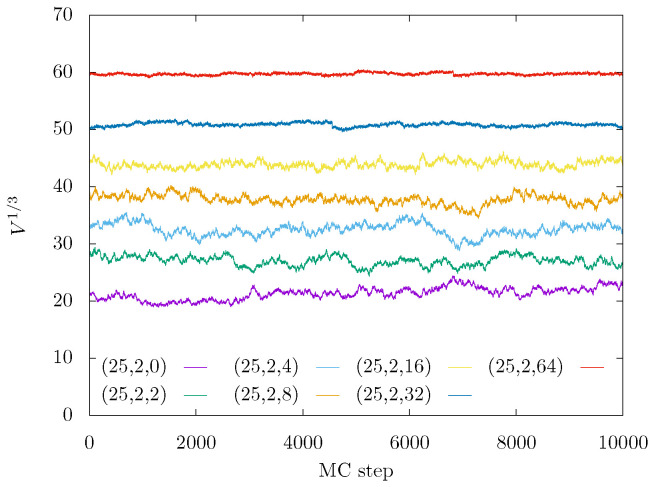
Traces of instantaneous box sizes during NPT Monte Carlo sampling for P=0 and gel architectural parameters (M,m,n), as indicated in the legend.

The simulations were performed in NPT ensemble by adding volume-changing moves [74]. The simulations started from extended conformations of gel backbone and coiled side-chains as corresponding to a good solvent. A thorough equlibration was performed for box sizes, energies, and end-to-end distances. The free (tuning) parameters of our HMC method were roughly set up according to optimal acceptance probability and the smallest integrated autocorrelation times. After equilibration (as illustrated by Figure 10), samples were accumulated, and standard deviations of their averages were estimated using the blocking method [75].

### 4.2. Molecular Dynamics Simulations

Each pair of the particles interact via the truncated Lennard–Jones interaction potential, which imposes strong repulsion between all particles at short distances:

(6) $$\begin{array}{c} U_{\mathrm{LJ}}\left(r\right)=\left\{\begin{array}{cc} 4\varepsilon \left({\left(\frac{\sigma }{r}\right)}^{12}-{\left(\frac{\sigma }{r}\right)}^{6}\right) & \mathrm{if}\;r<r_{\mathrm{cut}}\\ 0 & \mathrm{elsewhere} \end{array}\right., \end{array}$$

where *r* is the interparticle distance, σ=0.35 nm is a chosen characteristic size of the particles, $\varepsilon =k_{B}T$ is the depth of the potential, and $r_{\mathrm{cut}}$ is the cut-off distance beyond which the potential is set zero.

The bonds connecting the gel to a network are modeled using finite extension nonlinear elastic potential (FENE):

(7) $$U_{\mathrm{FENE}}\left(r\right)=-\frac{1}{2}K\Delta r_{\max}^{2}\ln\left[1-{\left(\frac{r-r_{0}}{\Delta r_{\max}}\right)}^{2}\right],$$

where *r* is the distance between the bonded segments, *K* is the magnitude of their interaction, $\Delta r_{\max}$ is the maximal stretching length of the bond, and $r_{0}$ is the equilibrium bond length. In our simulations we set $K=10kT/\sigma^{2}$, $\Delta r_{\max}=3\sigma$ and $r_{0}=0.0$ [76].

The Langevin thermostat [71] was used to guarantee the constant temperature of the system, T=300 K. The two additional terms to force in equation of motion were added.

(8) $$f_{i}=-\gamma v_{i}\left(t\right)+\sqrt{2\gamma kT}\eta_{i}\left(t\right),$$

where the first term corresponds to constant friction, with γ being a friction coefficient, and the second one corresponds to random thermal force, with $\eta_{i}$ being a normally distributed random vector; $v_{i}$—velocity of *i*-th particle, *t*—time.

In order to calculate the swelling ratio of hydrogel, we performed a series of simulations of the gel in a box of different volumes *V*. Each simulation results in a particular value of pressure *P*.

Our target observable is the free swelling equilibrium state, i.e., at the state where the applied to the gel pressure equals to zero. In order to localize this state, we first plotted P(V) dependence; then, using the least-squares method, we drew a line passing through the nearest to the *V* axis points. The place where this line crosses the *V*-axis defines the volume of free swelling equilibrium state.

## Figures and Tables

**Table 1:** Asymptotic power-law dependencies for normalized mesh size $R_{mesh}$, swelling ratio *Q*, and bulk osmotic modulus *G* in a free-swelling hairy gel with DP *M* of strands in good solvent (ν=3/5).

	$R_{\mathrm{mesh}}/{\mathrm{aM}}^{3/5}$	${\mathrm{QM}}^{-4/5}$	${\mathrm{Ga}}^{3}M^{9/5}/k_{B}T$
$C_{a}^{*}$	${\left(n/m\right)}^{9/25}$	${\left(n/m\right)}^{2/25}$	${\left(n/m\right)}^{-27/25}$
$C_{a}^{**}$	${\left(n/m\right)}^{9/25}$	${\left(n/m\right)}^{2/25}$	${\left(n/m\right)}^{-9/50}$
$C_{b}^{*}$	${\left(n/m\right)}^{3/10}m^{1/5}$	${\left(n/m\right)}^{-1/10}m^{3/5}$	${\left(n/m\right)}^{-9/10}m^{-3/5}$
$C_{b}^{**}$	$M^{2/5}$	$M^{6/5}{\left(n/m\right)}^{-1}$	$M^{-27/10}{\left(n/m\right)}^{9/4}$
